# Microbiological diagnostic performance of metagenomic next-generation sequencing compared with conventional culture for patients with community-acquired pneumonia

**DOI:** 10.3389/fcimb.2023.1136588

**Published:** 2023-03-16

**Authors:** Tianlai Lin, Xueliang Tu, Jiangman Zhao, Ling Huang, Xiaodong Dai, Xiaoling Chen, Yue Xu, Wushuang Li, Yaoyao Wang, Jingwei Lou, Shouxin Wu, Hongling Zhang

**Affiliations:** ^1^ Department of Intensive Care Unit, Quanzhou First Hospital Affiliated to Fujian Medical University, Quanzhou, China; ^2^ Shanghai Biotecan Pharmaceuticals Co., Ltd., Shanghai, China; ^3^ Shanghai Zhangjiang Institute of Medical Innovation, Shanghai, China; ^4^ Department of Clinical Laboratory, Huanghe Sanmenxia Hospital Affiliated to Henan University of Science and Technology, Sanmenxia, China

**Keywords:** metagenomic next-generation sequencing, culture, community-acquired pneumonia, conventional microbiological test, pathogen detection

## Abstract

**Background:**

Community-acquired pneumonia (CAP) is an extraordinarily heterogeneous illness, both in the range of responsible pathogens and the host response. Metagenomic next-generation sequencing (mNGS) is a promising technology for pathogen detection. However, the clinical application of mNGS for pathogen detection remains challenging.

**Methods:**

A total of 205 patients with CAP admitted to the intensive care unit were recruited, and broncho alveolar lavage fluids (BALFs) from 83 patients, sputum samples from 33 cases, and blood from 89 cases were collected for pathogen detection by mNGS. At the same time, multiple samples of each patient were tested by culture. The diagnostic performance was compared between mNGS and culture for pathogen detection.

**Results:**

The positive rate of pathogen detection by mNGS in BALF and sputum samples was 89.2% and 97.0%, which was significantly higher (*P* < 0.001) than that (67.4%) of blood samples. The positive rate of mNGS was significantly higher than that of culture (81.0% vs. 56.1%, *P* = 1.052e-07). A group of pathogens including *Mycobacterium abscessus*, *Chlamydia psittaci, Pneumocystis jirovecii, Orientia tsutsugamushi*, and *all viruses* were only detected by mNGS. Based on mNGS results, *Escherichia coli* was the most common pathogen (15/61, 24.59%) of non-severe patients with CAP, and *Mycobacterium tuberculosis* was the most common pathogen (21/144, 14.58%) leading to severe pneumonia. *Pneumocystis jirovecii* was the most common pathogen (26.09%) in severe CAP patients with an immunocompromised status, which was all detected by mNGS only.

**Conclusion:**

mNGS has higher overall sensitivity for pathogen detection than culture, BALF, and sputum mNGS are more sensitive than blood mNGS. mNGS is a necessary supplement of conventional microbiological tests for the pathogen detection of pulmonary infection.

## 1 Introduction

Infection is a common occurrence among patients in the intensive care unit (ICU) (Vincent et al., 2020), and community- acquired pneumonia (CAP) is the leading infectious disease cause of mortality worldwide (Torres et al., 2021). Severe CAP (SCAP) has a high mortality, and those survivors often have serious sequelae including the reduction of lung, mental, cognitive, and motor functions, especially those with an immunocompromised status (Sangla et al., 2020; Cilloniz et al., 2021). The identification of infected pathogens and prompt and adequate antimicrobial therapy are critical to improve the survival of patients with severe pneumonia (Cilloniz et al., 2021).

The conventional microbiological tests (CMTs) of an infectious disease diagnosis are mainly dependent on culture methodology combined with molecular detection through polymerase chain reaction (PCR) and the enzyme immunoassay (Miller et al., 2018). However, previous literature reported that no pathogen was detected in the majority of patients with pneumonia despite using comprehensive testing methods (Jain et al., 2015; Lu H. et al., 2022), resulting in delayed and inadequate treatment, prolonged stays, and increased mortality. Furthermore, empirical broad-spectrum antibiotic usage for patients without pathogen identification may lead to antimicrobial resistance.

Metagenomic next-generation sequencing (mNGS) is an unbiased approach that enables a broad identification of known and unexpected pathogens and has a special advantage on rare, novel, and atypical etiologies of complicated infectious diseases (Wang et al., 2019). In recent years, increasing studies have demonstrated its clinical value of sensitivity and speed in the pathogen detection of infectious diseases such as pneumonia (Chen et al., 2020; Wu et al., 2020; Peng et al., 2021; Sun et al., 2021). However, there are still limitations of mNGS for pathogen detection such as a high human host background, infection from colonization, and microbial contaminants limiting its sensitivity and specificity (Gu et al., 2019). On the other hand, it is still challenging for the interpretation of mNGS results, especially when they are inconsistent with clinical symptoms or CMT results. Thus, further investigation with a larger cohort across different geographical areas could promote the establishment of the clinical application standard of mNGS.

In this study, the results of the mNGS and conventional culture of 205 patients with CAP admitted to the ICU were retrospectively analyzed. This study aims to comprehensively evaluate and compare the diagnostic performance of mNGS and culture methods in patients with severe and non-severe CAP.

## 2 Methods

### 2.1 Study patients

A total of 205 patients with a diagnosis of community-acquired CAP admitted into the ICU were retrospectively included in this study from September 2019 to September 2021 in Quanzhou First Hospital. SCAP was defined as who included either one major criterion or no less than three minor criteria according to the Infectious Diseases Society of America/American Thoracic Society criteria (Metlay et al., 2019). The immunocompromised status was defined following the previous study (Wu et al., 2020). This study involving human participants was approved by the Ethical Committee of Quanzhou First Hospital (No. 2018220). Informed consent was obtained from each subject, and this study conforms to the ethical guidelines of the latest version of the Declaration of Helsinki.

The samples of broncho alveolar lavage fluid (BALF) from 83 patients, sputum samples from 33 cases, and blood from 89 cases were collected for pathogen detection by mNGS. At the same time, multiple samples of each patient were used for the CMT (Miller et al., 2018) such as microscopy with routine laboratory staining and the cultures of bacteria and fungi. The samples included BALF, sputum, blood, ascites, urine, and feces. Among 205 patients, 186 patients’ sample type of mNGS and the CMT was consistent.

### 2.2 Clinical data and treatment

The clinical data of each participant were collected from hospital electronic medical records combined with a questionnaire. Sequential Organ Failure Assessment (SOFA), Confusion, Urea, Respiratory Rate, Blood Pressure and Age Above or Below 65 Years (CURB-65) score, and Acute Physiologic Assessment and Chronic Health Evaluation II (APACHE II) scores were calculated to assess the severity degree and mortality risk in patients with pneumonia admitted to the ICU. The laboratory test results of blood routine examination were recorded including C-reactive protein, white blood cell and neutrophil count, serum creatinine, and blood lactic acid. Among 205 participants, 188 cases have received empirical antibiotic therapy prior to ICU admission. Then, the therapeutic regimens were adjusted based on the results of microbiology tests and mNGS combined with the clinical symptoms, imaging, and other infection indicators.

### 2.3 Sample collection and DNA extraction

At least 5 ml of BALF was collected by bronchoscopy following the standard clinical procedure (Chen et al., 2020) in a dry sterile tube. In a dry sterile tube, 1–3 ml of sputum was collected, which was liquefied with 0.1% dithiothreitol (DTT) at room temperature for 30 min before DNA extraction. Then, the DNA of BALF and sputum was extracted by the HostZEROTM Microbial DNA Kit (D4310, ZYMO RESEARCH) according to the kit instructions. No less than 5 ml of blood was collected using a cell-free DNA storage tube (CW2815M, CWBIO); then, plasma was separated by centrifugation at 1,600 g for 10 min. Cell-free DNA was extracted by the HiPure circulating DNA MIDI kit (D3182-03B, Magen). All samples were transported in drikold and cryopreserved until the experiment.

### 2.4 Metagenomic next-generation sequencing

The Kapa HyperPlus library preparation kit (kk8514, Kapa) was used to construct a library based on 1 ng DNA of each sample, following the manufacturer’s protocol. The length of the fragments of the library was analyzed by the Agilent 2100 Bioanalyzer, and the concentration of the library was controlled using the qubit dsDNA HS assay kit (Q32854, Thermo Fisher Scientific Inc.). The qualified library was loaded into the flow cell and sequenced on the NextSeq CN500 platform (Illumina). For each test, the negative control of no-template water was set to detect the contamination of the environment, reagent, and cross-sample in the process of the experiment. A historical positive sample was used as positive control. An internal control of specific molecular tags was put into sample to join in extraction to supervise the whole process of the experiment.

### 2.5 Bioinformatic analysis

Clean data were input into the follow-up analysis after removing the adapter, the low-quality and short reads, and reads with ≥10% N using Fastp software (v0.19.5) (Chen et al., 2018). Low-complexity reads were removed by Seqtk_sdust software (v1.3-r106). Then, human host sequences mapped to the human reference genome (GRCh38.p12) were subtracted using bowtie2 software (v 2.3.4.1). The remaining data were classified and annotated through alignment to four microbial genome databases consisting of bacteria, fungi, viruses, and parasites by Burrows–Wheeler Alignment software (v0.7.15). The databases were downloaded from the National Center for Biotechnology Information (NCBI) Refseq (ftp://ftp.ncbi.nlm.nih.gov/genomes/refseq/) including 17,822 microorganisms.

### 2.6 Criteria of positive metagenomic next-generation sequencing analysis results

The suspected background microorganisms from the microbial list were removed referring to the in-house background database. For the remaining microorganisms, the following criteria were used to identify infectious pathogens according to previous studies (Chen et al., 2020; Sun et al., 2021; Lu H. et al., 2022). Positive results were annotated if one of the following criteria were met. (1) For bacterium, the number of reads stringently mapped to pathogen species ≥50 or the suspected pathogens with reads<50 should be supported by a conventional culture result (Wang et al., 2019). (2) A fungus/mycoplasma/chlamydia/virus with at least three reads mapped to pathogen species, or supported by clinical culture (Chen et al., 2020). (3) *Mycobacterium tuberculosis* (MTB) with at least one read mapped to either the species or genus level due to the difficulty of DNA extraction and low possibility for contamination (Miao et al., 2018). (4) A non-tuberculous mycobacterium (NTM) was identified as a positive pathogen if the relative abundance of mapping reads in the genus or species level was in the top 10 of bacteria list, due to the balance of hospital-to-laboratory environmental contamination, which is commonly found in the environment (Miao et al., 2018). Mixed pulmonary infection was defined when two or more infectious pathogens were detected.

### 2.7 Statistical analysis

IBM SPSS Statistics 22 (IBM, NY, USA) was used for statistical analysis. R project (R 4.0.2, R Core Team; https://www.R-Project.org) and GraphPad Prism 6 (GraphPad Software, Inc., San Diego, CA, USA) were employed to plot graphics. Categorical variables were presented in the count number and percentage, which were compared with the chi-square test or Fisher exact test. Continuous variables were presented in mean ± standard deviation and were compared between two groups by a t-test if the data follow a normal distribution. If not, the median and interquartile range were presented and a non-parametric Mann–Whitney U test was performed. *P* < 0.05 indicated a statistically significant difference.

## 3 Results

### 3.1 Clinical characteristics of patients with community-acquired pneumonia

The demographic characteristics of patients in this study are shown in **Table 1**. Among 205 patients with CAP, 144 patients (70.24%) were diagnosed as SCAP, and 25 patients (12.2%) were immunocompromised. SCAP patients had elder age (*P* = 0.011) and a significant higher prevalence of an immunocompromised status (16.0% vs. 3.3%, *P* = 0.010) than that of non-severe CAP patients. 141 patients (68.8%) had a symptom of fever, and a higher ratio of patients with SCAP had a fever than patients with non-severe CAP patients (74.3% vs. 55.7%, *P*=0.009).

**Table 1:** Baseline characteristics and clinical manifestation of 205 patients with community-acquired pneumonia in the study on admission.

Clinical characteristics	Total	Non-severe CAP (n = 61)	SCAP (n = 144)	*P*- value
**Gender**				0.009
Male	164	42 (68.9%)	122 (84.7%)	
Female	41	19 (31.1%)	22 (15.3%)	
**Age (mean ± SD)**	65.41 ± 14.19	61.59 ± 14.92	67.06 ± 13.585	0.011
**BMI (mean ± SD)**	22.00 ± 2.97	22.05 ± 2.61	21.98 ± 3.12	0.876
**Smoking history**				0.060
No	135	46 (75.4%)	89 (61.8%)	
Yes	70	15 (24.6%)	55 (38.2%)	
**Drinking history**				0.785
No	167	49 (80.3%)	118 (81.9%)	
Yes	38	12 (19.7%)	26 (18.1%)	
**Immunocompromised**				0.010
No	180 (87.8%)	59 (96.7%)	121 (84.0%)	
Yes	25 (12.2%)	2 (3.3%)	23 (16.0)	
Underlying disease				
Cardiovascular diseases	111	31 (50.8%)	80 (55.6%)	0.534
Diabetes mellitus	50	18 (29.5)	32 (22.2%)	0.267
COPD	30	4 (6.6%)	26 (18.1%)	0.033
Malignant	30	7 (11.5%)	23 (16.0%)	0.405
**Fever**				0.009
No	64 (31.2%)	27 (44.3%)	37 (25.7%)	
Yes	141 (68.8%)	34 (55.7%)	107 (74.3%)	
Severity				
SOFA score	8 (5, 11))	7 (4, 11)	8 (5, 10.75)	0.755
CURB-65 score	3 (2, 3)	2 (1, 3)	3 (2, 4)	<0.001
APACHE II score	22 (16, 27)	20 (13.5, 26)	22 (16, 8)	0.098
WBC, *10^9/L	11.34 (7.02, 16.90)	11.83 (6.93, 17.56)	11.26 (7.12, 16.84)	0.900
<4*10^9/L	22	6 (9.8%)	16 (11.1%)	0.925
4–10*10^9/L	116	34 (55.7%)	82 (56.9%)	
>10*10^9/L	67	21 (34.4%)	46 (31.9%)	
Neutrophil, *10^9/L	10.87 (6.04, 18.38)	9.84 (4.99, 15.41)	11.55 (6.52, 22.45)	0.029
<1.8*10^9/L	14	7 (11.5%)	7 (4.9%)	0.066
1.8-6.3*10^9/L	149	38 (62.3%)	111 (77.1%)	
>6.3*10^9/L	42	16 (26.2%)	26 (18.1%)	
**CRP, mg/L**	86.87 (24.52, 142.32)	76.28 (16.68, 128.17)	96.62 (33.27, 149.97)	0.124
≤6 mg/L	22	9 (14.8%)	13 (9.0%)	0.226
>6 mg/L	131	52 (85.2%)	131 (91.0%)	

CAP, community-acquired pneumonia; SCAP, severe community-acquired pneumonia; COPD, chronic obstructive pulmonary disease; WBC, white blood cell; CRP, C-reactive protein; SOFA, Sequential Organ Failure Assessment; CURB-65, Confusion, Urea, Respiratory Rate, Blood Pressure and Age Above or Below 65 Years score; APACHE II Acute Physiologic Assessment and Chronic Health Evaluation II.

### 3.2 Comparison of positive rate between metagenomic next-generation sequencing and culture methods

**Figure 1:**
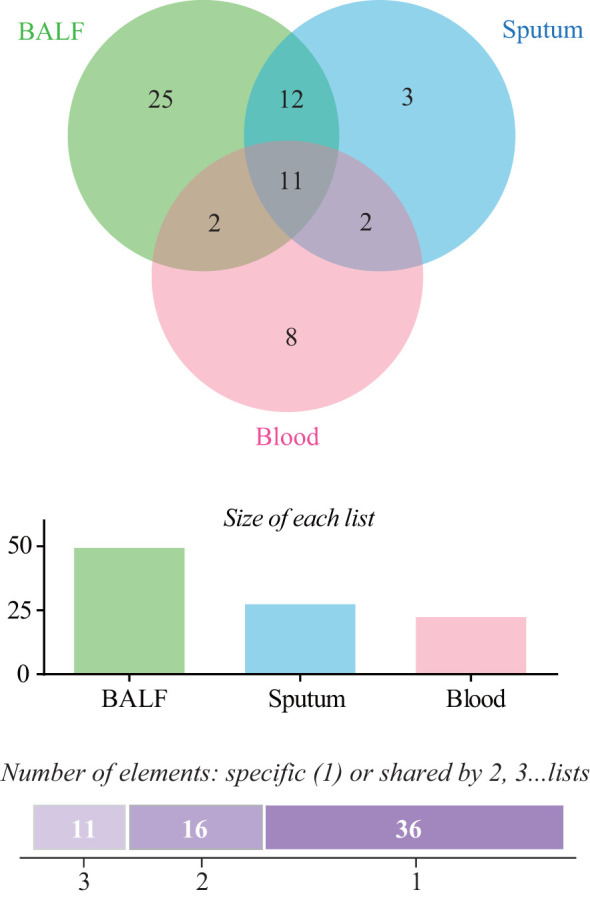
Comparison of pathogens detected by metagenomic next-generation sequencing (mNGS) among bronchoalveolar lavage fluid (BALF), sputum, and blood samples.

For mNGS technology, the sample type of 83 patients was BALF, 33 patients’ samples were sputum, and 89 patients’ samples were blood. Generally, a total of 50 pathogens were detected from the BALF samples of 83 patients, and 28 and 23 pathogens were detected from 33 sputum samples and 89 blood samples (**Figure 1**; **Supplementary Table 1**). The positive rate of pathogen detection in BALF and sputum samples was 89.2% and 97.0%, which was significantly higher (*P* < 0.001) than that (67.4%) of blood samples (**Table 2**).

**Table 2:** Positive rate of pathogens detected by metagenomic next-generation sequencing in different sample types involving bronchoalveolar lavage fluid, sputum, and blood.

Sample type	Total	Negative	Positive	P- value
BALF	93	9 (10.8%)	74 (89.2%)	<0.001
Sputum	33	1 (3.0%)	32 (97.0%)	
Blood	89	29 (32.6%)	60 (67.4%)	

BALF, bronchoalveolar lavage fluid.

**Figure 2:**
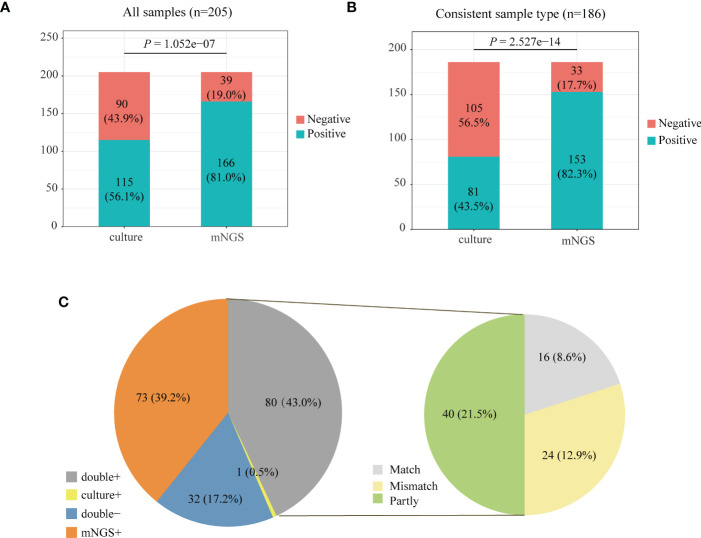
Positive rate comparison between metagenomic next-generation sequencing (mNGS) and laboratory culture for 205 community-acquired pneumonia (CAP) patients **(A)** and 186 pneumonia patients whose sample type was consistent for mNGS and culture **(B)**. **(C)** The positivity distribution of mNGS and culture for 186 patients with a consistent sample type. For the double-positive subgroup, the cases were divided into matched, mismatched, and partly matched groups based on the consistency of pathogens detected by the two methods.

We compared the positive rate of mNGS and culture methods, which is shown in **Figure 2A**. For all the 205 patients, the positive rate of mNGS was 81.0% which is much higher than that (56.1%) of culture results (*P* = 1.052e-07). Among 205 patients, 186 patients’ sample type of mNGS and culture was consistent. **Figure 2B** further compares the positive rate between mNGS and culture for samples with a consistent type, which also shows that the positive rate of mNGS was significantly higher than that of culture (82.3% vs. 43.5%, *P* = 2.527e−14).

### 3.3 Comparison of pathogen detection between metagenomic next-generation sequencing and culture

We analyzed the consistency of pathogens between mNGS and culture methods for 186 patients with a consistent sample type (**Figure 2C**). The results of mNGS and culture methods were both positive in 80 of 205 cases (43.0%) and both negative in 32 cases (17.2%). The results of 73 cases (39.2%) were only positive in mNGS, and one patient’s result was only positive by culture. To examine the results’ consistency of mNGS and culture, we further compared the detected pathogens between mNGS and culture for 80 patients whose mNGS result and culture result were both positive (**Figure 2C**, double+). The detected pathogens of mNGS were identical with the results of the culture method in 16 patients and partly matched with culture results in 40 patients. For 24 patients, the pathogens were mismatched between mNGS and culture methods (**Figure 2C**, right).

### 3.4 Pathogens’ profile of all CAP patients according to detection methods

**Figure 3:**
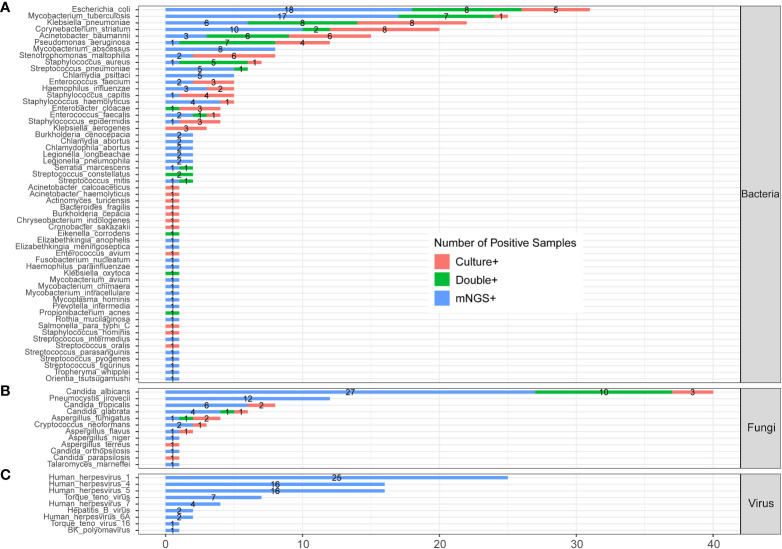
The comparison and overlap of infected pathogens between mNGS and laboratory culture in all 205 patients with CAP. **(A)** Bacteria levels; **(B)** fungi level; and **(C)** virus level.

**Figure 3** shows the pathogens’ profile of 205 CAP patients by mNGS and culture methods. The detected pathogens were divided into three kingdoms, namely, bacteria, fungi, and viruses. A total of 57 bacteria (**Figure 3A**), 12 fungi (**Figure 3B**), and 9 viruses (**Figure 3C**) were detected by mNGS and the CMT.

#### 3.4.1 Profile of bacteria

In the bacteria level (**Figure 3A**), *Escherichia coli* (*E. coli*) was the most common pathogen that was detected in 31 CAP patients. In addition, MTB (n=25), *Klebsiella pneumoniae* (n=22), *Corynebacterium striatum* (n=20), *Acinetobacter baumannii* (n=15), and *Pseudomonas aeruginosa* (n=12) were the common pathogens of CAP patients. A total of 23 bacteria were only detected by mNGS, including *Mycobacterium abscessus* (*M. abscessus*, n=8), *Chlamydia psittaci* (*C. psittaci*, n=5), *Burkholderia cenocepacia* (n=2), and *Chlamydia abortus* (n=2). A total of 12 bacteria were only detected by culture, including *Klebsiella aerogenes* (n=3), and *Acinetobacter calcoaceticus* (n=1). A total of 22 bacteria were detected by both mNGS and culture.

#### 3.4.2 Profile of fungi

In the fungi level (**Figure 3B**), *Candida albicans* (*C. albicans*) detected in 40 patients was the most frequent fungus, and 27 of cases were only detected by mNGS. *Pneumocystis jirovecii* (*P. jirovecii*) was the second common fungus detected in 12 cases by mNGS only.

#### 3.4.3 Profile of virus

All the nine viruses were all detected by the mNGS method. *Human herpesvirus* (HHV) was the most recurrent virus, including 25 cases of HHV-1, 16 cases of HHV-4, 16 cases of HHV -5, 4 cases of HHV-7, and 2 cases of HHV-6A (**Figure 3D**). The heatmap in **Supplementary Figure 1** shows the relative abundance of all nine viruses in 60 virus-infected patients.

We compared the results of the clinical culture test and mNGS from 186 CAP patients whose sample type was consistent between two methods, which is shown in **Supplementary Figure 2**. It had an overall consistency with the results of **Figure 3**.

### 3.5 Comparison of pathogens detected by metagenomic next-generation sequencing between severe and non-severe community-acquired pneumonia patients

**Figure 4:**
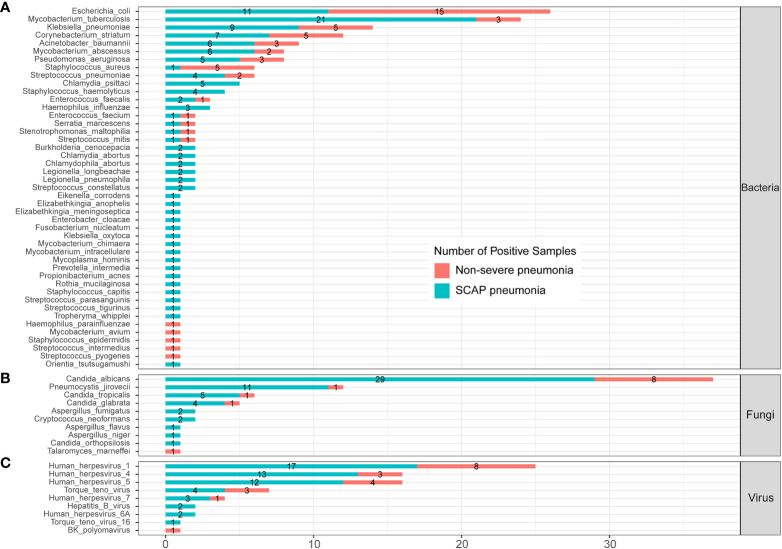
Infected pathogens detected by mNGS in severe and non-severe patients with CAP. **(A)** bacteria levels; **(B)** fungi level; **(C)** protozoa level; and (**C**) virus level.

To further evaluate the clinical significance of mNGS in patients with pulmonary infection, we compared the infected pathogens identified by mNGS between severe and non-severe CAP patients (**Figure 4**; **Supplementary Table 2**). A total of 45 bacteria were identified by mNGS in 205 CAP patients. There were 14 bacteria found in both severe and non-severe CAP patients, 5 bacteria were found only in infected non-severe patients, and 26 bacteria were only detected in SCAP patients (**Figure 4A**). *E. coli* is the most common infected bacteria in non-severe CAP patients (24.5%); the infection rate was significantly higher than that (7.64%) in the SCAP group (*P* = 0.001, **Supplementary Table 2**). MTB was the most common infected bacteria in SCAP patients (14.58%, **Supplementary Table 2**).

All five *C. psittaci* infected cases were from the SCAP group. From **Figure 3**, we can see that *C. psittaci* from five infected cases were all identified by mNGS only with negative culture results. In addition, all five *Staphylococcus haemolyticus (S. haemolyticus)–* infected cases belonged to the SCAP group. *S. haemolyticus* in four cases were identified by mNGS only, and one case was detected by culture. These results indicated that *C. psittaci* and *S. haemolyticus* were the common pathogens that had an almost entire probability leading to severe disease. mNGS has a very high clinical value on the identification of these two pathogens in SCAP patients with negative CMT results.

### 3.6 Comparison of pathogens between immunocompromised and immunocompetent patients with severe community-acquired pneumonia

**Figure 5:**
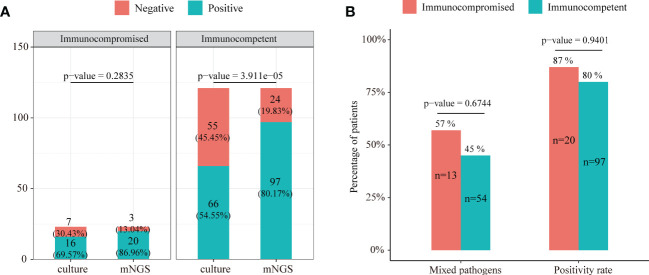
**(A)** The positive rate comparison between mNGS and the conventional test in immunocompetent and immunocompromised patients with severe pneumonia. **(B)** Comparison of the positive rate and ratio of mixed pathogen infection between immunocompetent and immunocompromised severe pneumonia patients.

The positive rates of mNGS and culture for both immunocompromised and immunocompetent patients with SCAP are illustrated in **Figure 5A**. The positive rate of mNGS in the immunocompetent group was significantly higher than that of culture (80.17% vs. 54.55%, *P* = 3.911e-05). However, there was no significant differences in the diagnostic positive rate between mNGS and culture in the immunocompromised group (86.96% vs. 69.57%, *P*=0.2835, **Figure 5A**). In addition, there was no significant difference in both the mixed pathogen rate (57% vs. 45%, *P* = 0.6744) and the diagnostic positive rate (87% vs. 80%, *P* = 0.9401) of mNGS between immunocompromised and immunocompetent patients with SCAP (**Figure 5B**).

**Figure 6:**
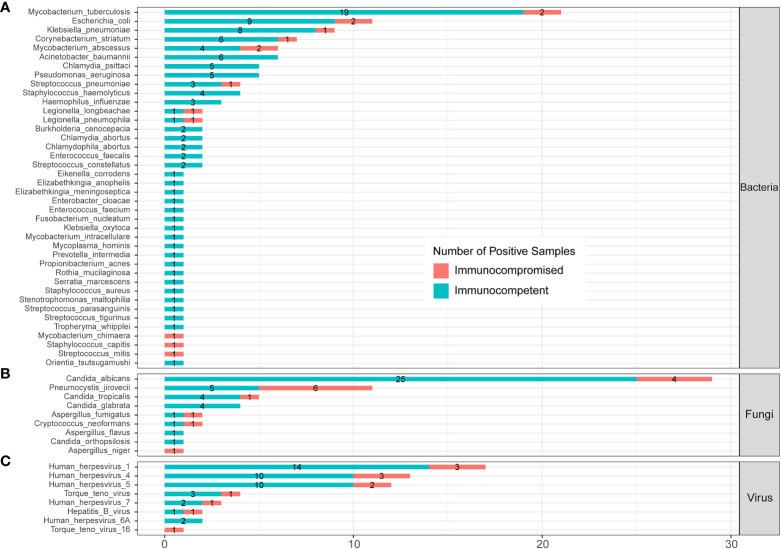
Infected pathogens detected by mNGS in immunocompetent and immunocompromised patients with severe pneumonia. **(A)** Bacteria levels; **(B)** fungi level; and **(C)** virus level.

The profile of infected pathogens identified by mNGS in 144 SCAP patients is shown in **Figure 6** and **Supplementary Table 3** according to patients with or without an immunocompromised status. A total of 40 bacteria were identified by mNGS from 144 SCAP patients. Among them, 29 bacteria were detected in immunocompetent cases only, 3 bacteria were detected in immunocompromised cases only, and 8 bacteria were found in both immunocompetent and immunocompromised groups. MTB was the most common infected bacterium both in immunocompetent (15.70%) and immunocompromised (8.70%) patients with SCAP. There was no significant difference in the positive rate of every bacterium between patients with or without an immunocompromised status (**Supplementary Table 3**).

A total of nine fungi were detected by mNGS in SCAP patients. Among them, three fungi were specifically identified in immunocompetent cases, one fungus was specifically detected in immunocompromised cases, and five fungi were found in both immunocompetent and immunocompromised groups. *C. albicans* was the most frequently infected fungus in the immunocompetent (20.66%) SCAP group. *P. jirovecii* was the most common pathogen (26.09%) in SCAP patients with an immunocompromised status. We found that the positive rate of *P. jirovecii* in immunocompromised patients with SCAP was obviously higher than that in immunocompetent cases (26.09% vs. 4.13%, *P*<0.001, **Supplementary Table 3**). All the *P. jirovecii–* infected patients were identified by mNGS only (**Figure 3**).

## 4 Discussion

In this study, we systematically compared the effectiveness of pathogen detection between mNGS and culture in a pairwise manner from 205 patients with CAP in the ICU. Firstly, we found that mNGS performed differently on pathogen detection for BALF, sputum, and blood samples. The mNGS of BALF and sputum samples had a higher sensitivity of pathogen detection than blood (*P* < 0.001), which was consistent with previous literature (Chen et al., 2020; Lu H. et al., 2022). Compared to sputum, BALF has the following advantages: reflecting the component at the level of alveoli and avoiding the contamination of oropharyngeal flora (Dubourg et al., 2015). Thus, BALF was suggested as the appropriate sample for the pathogen detection of SCAP patients because of its high sensitivity, good tolerance, and easily acquisition *via* bedside bronchoscopy (Hertz et al., 1991). Hence, if the BALF samples are not available for some patients with pneumonia, sputum is suggested as the second-rate choice. Xu Chen et al. (2020) reported that blood mNGS detected more viruses than BALF mNGS overall, but no similar phenomenon was observed in our study.

As the previous literature reported, our data also indicated that mNGS had overall higher sensitivity for pathogen detection compared with culture. In this study, MTB was the second most frequent pathogen (25/205, 12.2%) in the overall CAP cohort but the most common pathogen for the SCAP group. MTB is a main pathogen causing CAP in developing countries (Wei et al., 2020). A systematic review reported that more than 10% of patients with CAP in Asia were caused by MTB, which was consistent with our results (Peto et al., 2014). Previous literature showed that mNGS produced a similar sensitivity with Xpert and culture for MTB detection (Zhou et al., 2019; Shi et al., 2020). Some researchers suggested that the sensitivity of mNGS is not superior to that of culture for identifying common bacteria (excluding MTB and anaerobes) (Miao et al., 2018), and culture could identify the vast majority (74%) of bacterium-associated pneumonia (Toma et al., 2014). Our study showed mNGS had advantages over culture for MTB and NTM. For example, eight *M. abscessus*– infected patients were all diagnosed by mNGS only. *M. abscessus* is reported as the second most common non-tuberculous mycobacterial lung disease pathogen (Griffith and Daley, 2022), which is often regarded as one of the most antibiotic-resistant mycobacteria, leaving clinicians with few therapeutic options (Johansen et al., 2020). Thus, mNGS has a very high clinical value on the identification of MTB and NTM in patients with pulmonary infection, whose conventional microbiological tests give negative results.

In the cohort of our study, five patients with SCAP were identified as *C. psittaci* infection by mNGS. For all the five cases, culture gave the negative results. *C. psittaci* is an obligatory intracellular Gram-negative bacterium that typically infects birds; infections in humans mainly present as CAP (Hogerwerf et al., 2017). Conventional laboratory tests for *C. psittaci* include culture, a serological assay, and PCR. Culture is low efficient and requires a P3 facility (Balsamo et al., 2017); serological tests are only appropriate for a retrospective diagnosis (Tuuminen et al., 2000). Molecular detection by PCR is the specific and fastest method but needs the prejudgment of *C. psittaci* infection, which tends to be overlooked due to relatively low awareness by physicians. Our study accumulated evidence that mNGS is a useful tool to diagnose *C. psittaci* infection. Moreover, mNGS could even provide semiquantitative information (based on sequence reads) about the load of *C. psittaci*, which could be really important for judging whether it is the causative pathogen in mixed infected samples (Gu et al., 2020). CY Kong et al.’s study (Kong et al., 2021) suggested that poultry exposure history, high fever, elevated inflammatory biomarkers, and elevated lactate dehydrogenase, combined with air-containing bronchial shadow consolidation with little or no secretions, may guide the early clinical diagnosis of *C. psittaci* pneumonia.

In this study, *P. jirovecii* was the most common pathogen in SCAP patients with an immunocompromised status, whose infected frequency was significantly higher than that of immunocompetent patients with SCAP. Previous researchers reported that the asymptomatic lung colonization of *P. jirovecii* could occur in people with normal immune systems (Truong and Ashurst, 2022). They may unknowingly become asymptomatic carriers for the spread of *Pneumocystis* to immunocompromised individuals through an airborne route (Truong and Ashurst, 2022). Patients with *P. jirovecii* pneumonia may have the symptoms of fever, cough, dyspnea, and respiratory failure in severe cases. The diagnosis of *P. jirovecii* pneumonia is multifactorial and may include the laboratory tests of BALF and sputum, chest radiograph, chest computed tomography, or lung biopsies (Langevin and Saleh, 2016). A definitive diagnosis of *P. jirovecii* pneumonia requires identifying the organism by the PCR of respiratory specimens, dye staining, or fluorescein antibody staining (Limper et al., 1989). Since *Pneumocystis* cannot be cultured, in this study, all patients infected with *P. jirovecii* were diagnosed by mNGS. Our study demonstrated that mNGS could quickly and accurately diagnose *P. jirovecii* pneumonia, which was also approved by other reseachers (Liu et al., 2021; Lu X. et al., 2022; Sun et al., 2022; Wang et al., 2022; Zhao et al., 2022). A combination of clinical symptoms, laboratory testing, and imaging examination is suggested to make a comprehensive judgment along with the mNGS test.

There are some limitations in our study. Firstly, mNGS in this study was only performed on the DNA level without concomitant RNA-seq data. It will miss the RNA virus and microbial transcriptome information. Comprehensive mNGS- based DNA-seq combined with RNA-seq could further improve the sensitivity of pathogen detection. In addition, the sample types were varied including BALF, sputum, and blood, and the detection efficiency of virus sample types was different. The lack of a consistent sample- collecting method and site affected the results of mNGS. To further comprehensively evaluate the application value of mNGS in the diagnosis of pulmonary infections, multicenter prospective studies with a larger number of participants are required. Further research is needed to evaluate the possible improvement in clinical outcomes and the cost-effectiveness of mNGS for infectious diseases.

## Conclusions

In conclusion, this study demonstrated that mNGS had higher overall sensitivity for the pathogen identification of pulmonary infections than culture. BALF and sputum mNGS had advantage over blood mNGS in pathogen detection. mNGS is superior in detecting MTB, NTM, viruses, *P. jirovecii*, and chlamydia. Thus, mNGS is a necessary complement of conventional microbiological tests and helps clinicians to make treatment decisions for patients with an unknown clinical diagnosis.

## Data availability statement

The original contributions presented in the study are publicly available. This data can be found here: NCBI SRA under project ID: PRJNA917446.

## Ethics statement

The studies involving human participants were reviewed and approved by the Ethical Committee of Quanzhou First Hospital. The patients/participants provided their written informed consent to participate in this study.

## Author contributions

TL, JL, and HZ designed the project. LH, XD, and XC collected samples and clinical data. YX, WL, and YW performed experiments and analyzed the data. TL, JZ, and XT wrote the manuscript. All authors read and approved the final manuscript.
