# Erratum: Microbiological diagnostic performance of metagenomic next-generation sequencing compared with conventional culture for patients with community-acquired pneumonia

**DOI:** 10.3389/fcimb.2023.1233180

**Published:** 2023-06-14

**Authors:** Frontiers Production Office

**Affiliations:** Frontiers Media SA, Lausanne, Switzerland

**Keywords:** metagenomic next-generation sequencing, culture, community-acquired pneumonia, conventional microbiological test, pathogen detection

Due to a production error, the Data Availability Statement that was originally published was incorrect.

The Data Availability Statement should be:

“The original contributions presented in the study are publicly available. This data can be found here: NCBI SRA under project ID: PRJNA917446.”

The publisher apologizes for this mistake.

The original version of this article has been updated.

