# Corrigendum: Microbiological diagnostic performance of metagenomic next-generation sequencing compared with conventional culture for patients with community-acquired pneumonia

**DOI:** 10.3389/fcimb.2023.1205802

**Published:** 2023-08-14

**Authors:** Tianlai Lin, Xueliang Tu, Jiangman Zhao, Ling Huang, Xiaodong Dai, Xiaoling Chen, Yue Xu, Wushuang Li, Yaoyao Wang, Jingwei Lou, Shouxin Wu, Hongling Zhang

**Affiliations:** ^1^ Department of Intensive Care Unit, Quanzhou First Hospital Affiliated to Fujian Medical University, Quanzhou, China; ^2^ Shanghai Biotecan Pharmaceuticals Co., Ltd., Shanghai, China; ^3^ Shanghai Zhangjiang Institute of Medical Innovation, Shanghai, China; ^4^ Department of Clinical Laboratory, Huanghe Sanmenxia Hospital Affiliated to Henan University of Science and Technology, Sanmenxia, China

**Keywords:** metagenomic next-generation sequencing, culture, community-acquired pneumonia, conventional microbiological test, pathogen detection

**Error in Figure/Table Legend**

In the published article, there was an error in the legend for **Figure 3** as published. “(C) protozoa level” was included in legend of **Figure 3**, which should be deleted. The corrected legend appears below.

**Figure 3** The comparison and overlap of infected pathogens between mNGS and laboratory culture in all 205 patients with CAP. **(A)** Bacteria levels; **(B)** fungi level; and **(C)** virus level.

In the published article, there was an error in the legend for **Figure 4** as published. “(C) protozoa level” was included in legend of **Figure 4**, which should be deleted. The corrected legend appears below.

**Figure 4** Infected pathogens detected by mNGS in severe and non-severe patients with CAP. **(A)** bacteria levels; **(B)** fungi level; and **(C)** virus level.

In the published article, there was an error in the legend for **Figure 6** as published. “(C) protozoa level” was included in legend of **Figure 6**, which should be deleted. The corrected legend appears below.

**Figure 6** Infected pathogens detected by mNGS in immunocompetent and immunocompromised patients with severe pneumonia. **(A)** Bacteria levels; **(B)** fungi level; and **(C)** virus level.

**Error in Figure/Table**

In the published article, there was an error in **Figure 3** as published. *Orientia tsutsugamushi* was divided in Part C “Protozoa”, which should be moved to Part A “Bacteria”. The corrected **Figure 3** and its caption ** The comparison and overlap of infected pathogens between mNGS and laboratory culture in all 205 patients with CAP. (A) Bacteria levels; (B) fungi level; and (C) virus level. appear below.

**Figure 3:**
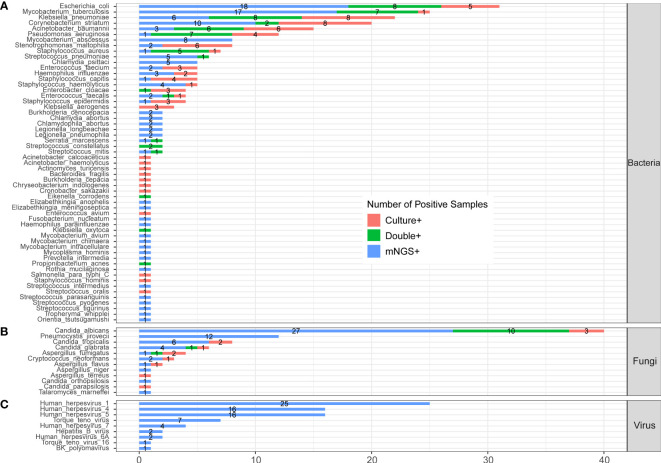
The comparison and overlap of infected pathogens between mNGS and laboratory culture in all 205 patients with CAP. **(A)** Bacteria levels; **(B)** fungi level; and **(C)** virus level.

In the published article, there was an error in **Figure 4** as published. *Orientia tsutsugamushi* was divided in Part C “Protozoa”, which should be moved to Part A “Bacteria”. The corrected **Figure 4** and its caption ** Infected pathogens detected by mNGS in severe and non-severe patients with CAP. (A) bacteria levels; (B) fungi level; and (C) virus level. appear below.

**Figure 4:**
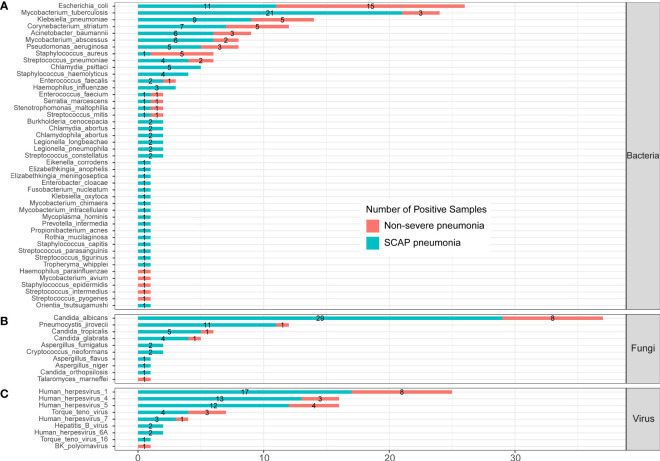
Infected pathogens detected by mNGS in severe and non-severe patients with CAP. **(A)** bacteria levels; **(B)** fungi level; and **(C)** virus level.

In the published article, there was an error in **Figure 6** as published. *Orientia tsutsugamushi* was divided in Part C “Protozoa”, which should be moved to Part A “Bacteria”. The corrected **Figure 6** and its caption ** Infected pathogens detected by mNGS in immunocompetent and immunocompromised patients with severe pneumonia. (A) Bacteria levels; (B) fungi level; and (C) virus level. appear below.

**Figure 6:**
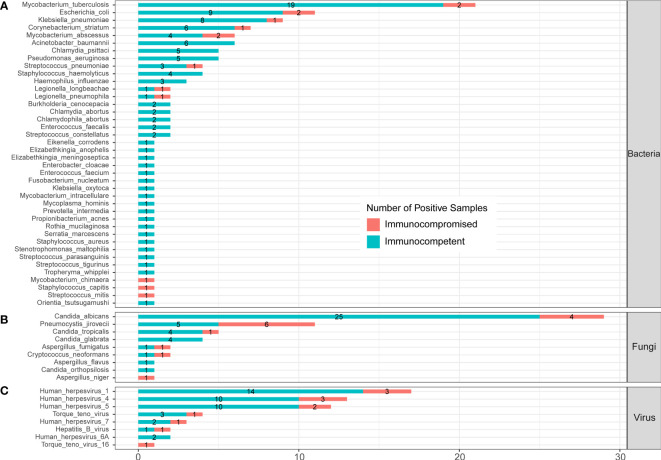
Infected pathogens detected by mNGS in immunocompetent and immunocompromised patients with severe pneumonia. **(A)** Bacteria levels; **(B)** fungi level; and **(C)** virus level.

**Incorrect Supplementary Material**

In the published article, there was an error in Supplementary Figure 2. *Orientia tsutsugamushi* was divided in Part C “Protozoa”, which should be moved to Part A “Bacteria”.

**Supplementary Figure 2.** The comparison and overlap of infected pathogens between metagenomic next-generation sequencing (mNGS) and laboratory culture in 186 patients with CAP whose sample type was consistent between two methods. **(A)** Bacteria levels; **(B)** Fungi level; **(C)** Virus level.

In the published article, there was an error in Supplementary Table 2. *Orientia tsutsugamushi* was divided in Part of “Protozoa”, which should be moved to Part of “Bacteria”.

**Supplementary Table 2.** Comparison of pathogens detected by mNGS between non-severe CAP and SCAP groups.

In the published article, there was an error in Supplementary Table 3. *Orientia tsutsugamushi* was divided in Part of “Protozoa”, which should be moved to Part of “Bacteria”.

**Supplementary Table 3.** Comparison of pathogens detected by mNGS between immunocompetent and immunocompromised patients with SCAP.

**Text Correction**

In the published article, there was an error. *Orientia tsutsugamushi* was wrongly be categorized as Protozoa, which should be a bacterium. So, the number of detected bacteria and protozoan should be corrected.

A correction has been made to **3 Results**, *3.4 Pathogens’ profile of all CAP patients according to detection methods*, Paragraph 1. This sentence previously stated:

“The detected pathogens were divided into four kingdoms, namely, bacteria, fungi, protozoa, and viruses. A total of 56 bacteria (**Figure 3A**), 12 fungi (**Figure 3B**), 1 protozoan (**Figure 3C**), and 9 viruses (**Figure 3D**) were detected by mNGS and the CMT.”

The corrected sentence appears below:

“The detected pathogens were divided into three kingdoms, namely, bacteria, fungi, and viruses. A total of 57 bacteria (**Figure 3A**), 12 fungi (**Figure 3B**), and 9 viruses (**Figure 3C**) were detected by mNGS and the CMT.”

In the published article, there was an error. *Orientia tsutsugamushi* was wrongly be categorized as Protozoa, which should be a bacterium. So, the number of detected bacteria and protozoan should be corrected.

A correction has been made to **3 Results**, *3.4.1 Profile of bacteria*, Paragraph 1. This sentence previously stated:

“A total of 22 bacteria were only detected by mNGS, including *Mycobacterium abscessus* (*M. abscessus*, n=8), *Chlamydia psittaci* (*C. psittaci*, n=5), *Burkholderia cenocepacia* (n=2), and *Chlamydia abortus* (n=2).”

The corrected sentence appears below:

“A total of 23 bacteria were only detected by mNGS, including *Mycobacterium abscessus* (*M. abscessus*, n=8), *Chlamydia psittaci* (*C. psittaci*, n=5), *Burkholderia cenocepacia* (n=2), and *Chlamydia abortus* (n=2).”

In the published article, there was an error. *Orientia tsutsugamushi* was wrongly be categorized as Protozoa, which should be a bacterium. So, result of protozoa should be deleted.

A correction has been made to **3 Results**, *3.4.2 Profile of fungi and protozoa*, sub-title and Paragraph 1. This paragraph previously stated:

“3.4.2 Profile of fungi and protozoa

In the fungi level (**Figure 3B**), *Candida albicans* (*C. albicans*) detected in 40 patients was the most frequent fungus, and 27 of cases were only detected by mNGS. *Pneumocystis jirovecii* (*P. jirovecii*) was the second common fungus detected in 12 cases by mNGS only. One case was positive of *Orientia tsutsugamushi*, which was detected by mNGS only (**Figure 3C**).”

The corrected paragraph appears below:

“3.4.2 Profile of fungi

In the fungi level (**Figure 3B**), *Candida albicans* (*C. albicans*) detected in 40 patients was the most frequent fungus, and 27 of cases were only detected by mNGS. *Pneumocystis jirovecii* (*P. jirovecii*) was the second common fungus detected in 12 cases by mNGS only.”

In the published article, there was an error. *Orientia tsutsugamushi* was wrongly be categorized as Protozoa, which should be a bacterium. So, the number of detected bacteria should be corrected.

A correction has been made to **3 Results**, *3.5 Comparison of pathogens detected by metagenomic next-generation sequencing between severe and non-severe community-acquired pneumonia patients*, Paragraph 1. These sentences previously stated:

“A total of 44 bacteria were identified by mNGS in 205 CAP patients. There were 14 bacteria found in both severe and non-severe CAP patients, 5 bacteria were found only in infected non-severe patients, and 25 bacteria were only detected in SCAP patients (**Figure 4A**).”

The corrected sentence appears below:

“A total of 45 bacteria were identified by mNGS in 205 CAP patients. There were 14 bacteria found in both severe and non-severe CAP patients, 5 bacteria were found only in infected non-severe patients, and 26 bacteria were only detected in SCAP patients (**Figure 4A**).”

In the published article, there was an error. *Orientia tsutsugamushi* was wrongly be categorized as Protozoa, which should be a bacterium. So, the number of detected bacteria should be corrected.

A correction has been made to **3 Results**, *3.6 Comparison of pathogens between immunocompromised and immunocompetent patients with severe community-acquired pneumonia*, Paragraph 2. These sentences previously stated:

“A total of 39 bacteria were identified by mNGS from 144 SCAP patients. Among them, 28 bacteria were detected in immunocompetent cases only, 3 bacteria were detected in immunocompromised cases only, and 8 bacteria were found in both immunocompetent and immunocompromised groups.”

The corrected sentence appears below:

“A total of 40 bacteria were identified by mNGS from 144 SCAP patients. Among them, 29 bacteria were detected in immunocompetent cases only, 3 bacteria were detected in immunocompromised cases only, and 8 bacteria were found in both immunocompetent and immunocompromised groups.”

In the published article, there was an error. *Orientia tsutsugamushi* was wrongly be categorized as Protozoa, which should be a bacterium. So, the word “Protozoa” should be deleted in Conclusions.

A correction has been made to **Conclusions**. These sentences previously stated:

“mNGS is superior in detecting MTB, NTM, viruses, *P. jirovecii*, chlamydia, and protozoa.”

The corrected sentence appears below:

“mNGS is superior in detecting MTB, NTM, viruses, *P. jirovecii*, and chlamydia.”

The authors apologize for these errors and state that this do not change the scientific conclusions of the article in any way. The original article has been updated.

