# Supplementary material for: Microbiological diagnostic performance of metagenomic next-generation sequencing compared with conventional culture for patients with community-acquired pneumonia
Source: Front Cell Infect Microbiol. 2023 Mar 16;13:1136588. doi: 10.3389/fcimb.2023.1136588 (PMC10061305; doi:10.3389/fcimb.2023.1136588)
Supplement: Supplementary file 1 [file DataSheet_1.docx]

Supplementary Material

Microbiological diagnostic performance of metagenomic next-generation sequencing compared with conventional culture for patients with community-acquired pneumonia

Tianlai Lin^1†^, Xueliang Tu^2†^, Jiangman Zhao^3,4†^, Ling Huang^1^, Xiaodong Dai^1^, Xiaoling Chen^1^, Yue Xu^3,4^, Wushuang Li^3,4^, Yaoyao Wang^3,4^, Jingwei Lou ^3,4*^, Shouxin Wu^3,4*^ and Hongling Zhang^1*^

*** Correspondence:** Jingwei Lou: [jingweilou@biotecan.com](mailto:jingweilou@biotecan.com); Shouxin Wu: [swu@biotecan.com](mailto:swu@biotecan.com); Hongling Zhang: [zhanghonglingysj@163.com](mailto:zhanghonglingysj@163.com)

# Supplementary Figures and Tables

## Supplementary Figures

#
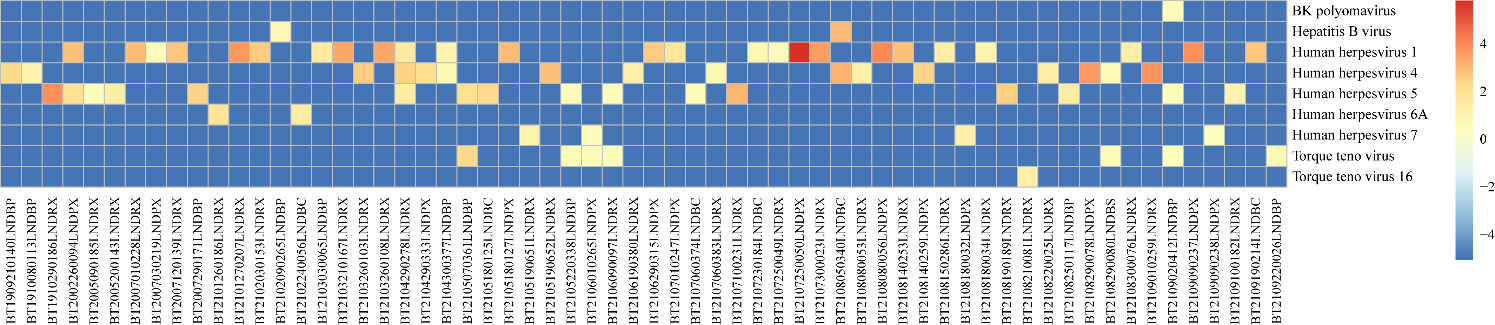


# Supplementary Figure 1. Heatmap of pathogens detected by mNGS according to number of sequencing reads.


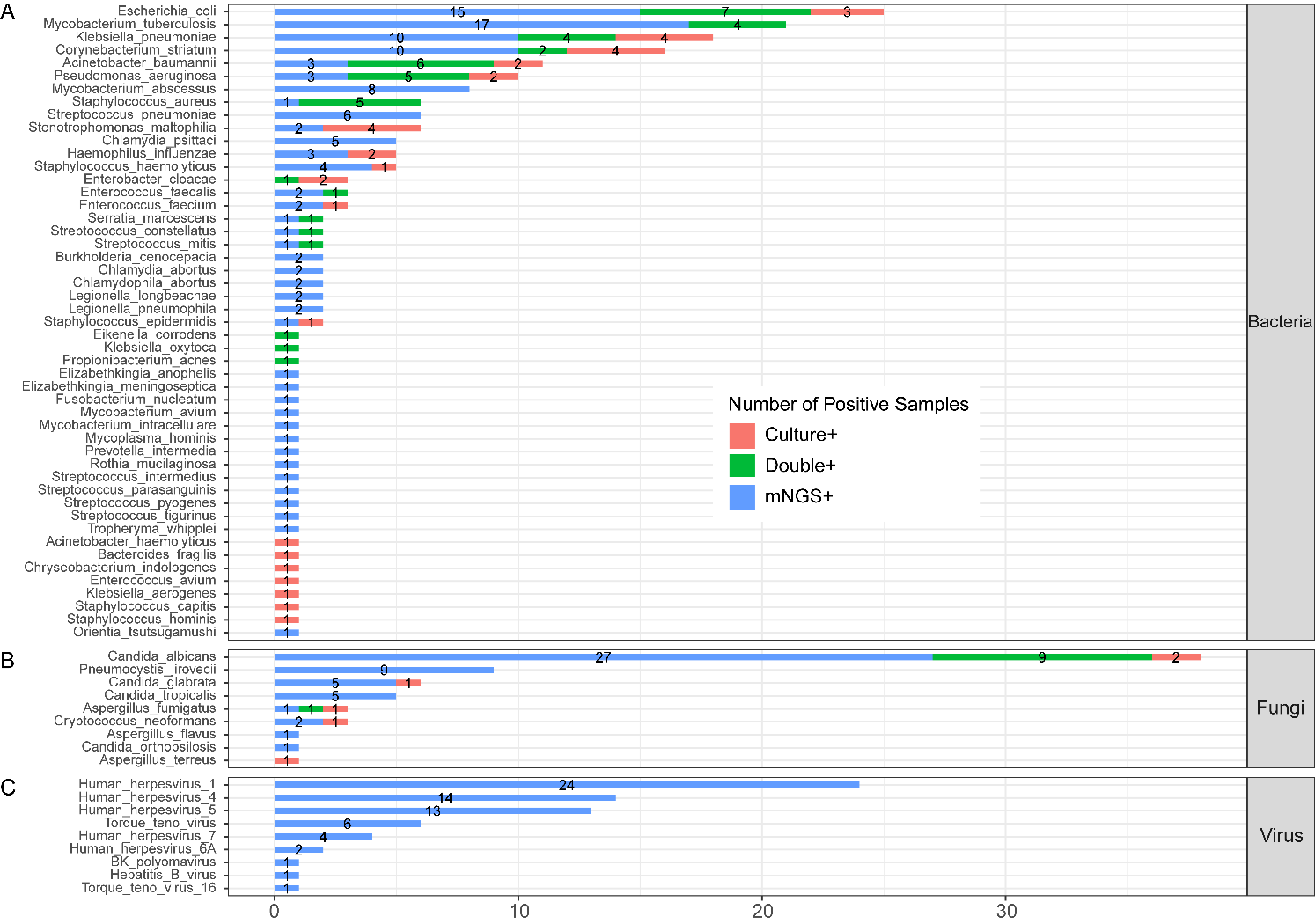


**Supplementary Figure 2.** The comparison and overlap of infected pathogens between metagenomic next-generation sequencing (mNGS) and laboratory culture in 186 patients with CAP whose sample type was consistent between two methods. **(A)** Bacteria levels; **(B)** Fungi level; **(C)** Virus level.

## Supplementary Tables

**Supplementary Table 1.** The pathogens detected by mNGS in BALF, sputum and blood samples.

| **Pathogens** | **Pathogens detected in BALF** | **Pathogens detected in sputum** | **Pathogens detected in blood** |
| --- | --- | --- | --- |
| Human herpesvirus 5 | Human herpesvirus 5 | Human herpesvirus 5 | Human herpesvirus 5 |
| Mycobacterium chimaera | Mycobacterium chimaera | / | / |
| Aspergillus niger | Aspergillus niger | / | / |
| Candida tropicalis | Candida tropicalis | Candida tropicalis | / |
| Pneumocystis jirovecii | Pneumocystis jirovecii | Pneumocystis jirovecii | Pneumocystis jirovecii |
| Candida albicans | Candida albicans | Candida albicans | Candida albicans |
| Mycobacterium abscessus | Mycobacterium abscessus | Mycobacterium abscessus | / |
| Mycobacterium avium | Mycobacterium avium | / | / |
| Candida glabrata | Candida glabrata | Candida glabrata | / |
| Streptococcus tigurinus | Streptococcus tigurinus | / | / |
| Human herpesvirus 1 | Human herpesvirus 1 | Human herpesvirus 1 | Human herpesvirus 1 |
| Candida orthopsilosis | Candida orthopsilosis | / | / |
| Elizabethkingia meningoseptica | Elizabethkingia meningoseptica | / | / |
| Elizabethkingia anophelis | Elizabethkingia anophelis | / | / |
| Burkholderia cenocepacia | Burkholderia cenocepacia | / | / |
| Mycobacterium tuberculosis | Mycobacterium tuberculosis | Mycobacterium tuberculosis | Mycobacterium tuberculosis |
| Klebsiella pneumoniae | Klebsiella pneumoniae | Klebsiella pneumoniae | Klebsiella pneumoniae |
| Chlamydophila abortus | Chlamydophila abortus | / | / |
| Chlamydia psittaci | Chlamydia psittaci | Chlamydia psittaci | / |
| Human herpesvirus 6A | Human herpesvirus 6A | / | / |
| Acinetobacter baumannii | Acinetobacter baumannii | Acinetobacter baumannii | / |
| Corynebacterium striatum | Corynebacterium striatum | Corynebacterium striatum | / |
| Eikenella corrodens | Eikenella corrodens | / | / |
| Fusobacterium nucleatum | Fusobacterium nucleatum | / | / |
| Prevotella intermedia | Prevotella intermedia | / | / |
| Enterococcus faecalis | Enterococcus faecalis | / | Enterococcus faecalis |
| Staphylococcus haemolyticus | Staphylococcus haemolyticus | Staphylococcus haemolyticus | / |
| Human herpesvirus 4 | Human herpesvirus 4 | Human herpesvirus 4 | Human herpesvirus 4 |
| Serratia marcescens | Serratia marcescens | Serratia marcescens | / |
| Streptococcus mitis | Streptococcus mitis | / | Streptococcus mitis |
| Human herpesvirus 7 | Human herpesvirus 7 | Human herpesvirus 7 | / |
| Mycobacterium intracellulare | Mycobacterium intracellulare | / | / |
| Escherichia coli | Escherichia coli | Escherichia coli | Escherichia coli |
| Torque teno virus | Torque teno virus | Torque teno virus | Torque teno virus |
| Chlamydia abortus | Chlamydia abortus | / | / |
| Aspergillus fumigatus | Aspergillus fumigatus | / | / |
| Haemophilus influenzae | Haemophilus influenzae | Haemophilus influenzae | / |
| Enterococcus faecium | Enterococcus faecium | Enterococcus faecium | / |
| Stenotrophomonas maltophilia | Stenotrophomonas maltophilia | / | / |
| Staphylococcus aureus | Staphylococcus aureus | Staphylococcus aureus | Staphylococcus aureus |
| Streptococcus parasanguinis | Streptococcus parasanguinis | / | / |
| Cryptococcus neoformans | Cryptococcus neoformans | Cryptococcus neoformans | / |
| Torque teno virus 16 | Torque teno virus 16 | / | / |
| Mycoplasma hominis | Mycoplasma hominis | / | / |
| Rothia mucilaginosa | Rothia mucilaginosa | / | / |
| Streptococcus pyogenes | Streptococcus pyogenes | / | / |
| Streptococcus intermedius | Streptococcus intermedius | / | / |
| Tropheryma whipplei | Tropheryma whipplei | / | / |
| Pseudomonas aeruginosa | Pseudomonas aeruginosa | Pseudomonas aeruginosa | Pseudomonas aeruginosa |
| Aspergillus flavus | Aspergillus flavus | / | / |
| Streptococcus pneumoniae | / | Streptococcus pneumoniae | Streptococcus pneumoniae |
| Enterobacter cloacae | / | Enterobacter cloacae | / |
| Staphylococcus capitis | / | Staphylococcus capitis | / |
| Staphylococcus epidermidis | / | Staphylococcus epidermidis | / |
| Legionella longbeachae | / | Legionella longbeachae | Legionella longbeachae |
| Klebsiella oxytoca | / | / | Klebsiella oxytoca |
| Legionella pneumophila | / | / | Legionella pneumophila |
| Hepatitis B virus | / | / | Hepatitis B virus |
| Haemophilus parainfluenzae | / | / | Haemophilus parainfluenzae |
| Talaromyces marneffei | / | / | Talaromyces marneffei |
| BK polyomavirus | / | / | BK polyomavirus |
| Streptococcus constellatus | / | / | Streptococcus constellatus |
| Orientia tsutsugamushi | / | / | Orientia tsutsugamushi |

**Supplementary Table 2.** Comparison of pathogens detected by mNGS between non-severe CAP and SCAP groups.

| **Infected pathogens** | **Total** | **SCAP** | **Non-severe CAP** | ***P* value** |
| --- | --- | --- | --- | --- |
|  |  | **(n=144)** | **(n=61)** |  |
| **Bacteria** |  |  |  |  |
| Escherichia_coli | 26 | 11 (7.64%) | 15 (24.59%) | **0.001** |
| Mycobacterium_tuberculosis | 24 | 21 (14.58%) | 3 (4.92%) | 0.058 |
| Klebsiella_pneumoniae | 14 | 9 (6.25%) | 5 (8.20%) | 0.613 |
| Corynebacterium_striatum | 12 | 7 (4.86%) | 5 (8.20%) | 0.352 |
| Acinetobacter_baumannii | 9 | 6 (4.17%) | 3 (4.92%) | 0.727 |
| Mycobacterium_abscessus | 8 | 6 (4.17%) | 2 (3.28%) | 1.000 |
| Pseudomonas_aeruginosa | 8 | 5 (3.47%) | 3 (4.92%) | 0.697 |
| Staphylococcus_aureus | 6 | 1 (0.69%) | 5 (8.20%) | **0.010** |
| Streptococcus_pneumoniae | 6 | 4 (2.78%) | 2 (3.28%) | 1.000 |
| Chlamydia_psittaci | 5 | 5 (3.47%) | 0 (0%) | 0.325 |
| Staphylococcus_haemolyticus | 4 | 4 (2.78%) | 0 (0%) | 0.320 |
| Enterococcus_faecalis | 3 | 2 (1.39%) | 1 (1.64%) | 1.000 |
| Haemophilus_influenzae | 3 | 3 (2.08%) | 0 (0%) | 0.556 |
| Enterococcus_faecium | 2 | 1 (0.69%) | 1 (1.64%) | 0.508 |
| Serratia_marcescens | 2 | 1 (0.69%) | 1 (1.64%) | 0.508 |
| Stenotrophomonas_maltophilia | 2 | 1 (0.69%) | 1 (1.64%) | 0.508 |
| Streptococcus_mitis | 2 | 1 (0.69%) | 1 (1.64%) | 0.508 |
| Burkholderia_cenocepacia | 2 | 2 (1.39%) | 0 (0%) | 1.000 |
| Chlamydia_abortus | 2 | 2 (1.39%) | 0 (0%) | 1.000 |
| Chlamydophila_abortus | 2 | 2 (1.39%) | 0 (0%) | 1.000 |
| Legionella_longbeachae | 2 | 2 (1.39%) | 0 (0%) | 1.000 |
| Legionella_pneumophila | 2 | 2 (1.39%) | 0 (0%) | 1.000 |
| Streptococcus_constellatus | 2 | 2 (1.39%) | 0 (0%) | 1.000 |
| Eikenella_corrodens | 1 | 1 (0.69%) | 0 (0%) | 1.000 |
| Elizabethkingia_anophelis | 1 | 1 (0.69%) | 0 (0%) | 1.000 |
| Elizabethkingia_meningoseptica | 1 | 1 (0.69%) | 0 (0%) | 1.000 |
| Enterobacter_cloacae | 1 | 1 (0.69%) | 0 (0%) | 1.000 |
| Fusobacterium_nucleatum | 1 | 1 (0.69%) | 0 (0%) | 1.000 |
| Klebsiella_oxytoca | 1 | 1 (0.69%) | 0 (0%) | 1.000 |
| Mycobacterium_chimaera | 1 | 1 (0.69%) | 0 (0%) | 1.000 |
| Mycobacterium_intracellulare | 1 | 1 (0.69%) | 0 (0%) | 1.000 |
| Mycoplasma_hominis | 1 | 1 (0.69%) | 0 (0%) | 1.000 |
| Prevotella_intermedia | 1 | 1 (0.69%) | 0 (0%) | 1.000 |
| Propionibacterium_acnes | 1 | 1 (0.69%) | 0 (0%) | 1.000 |
| Rothia_mucilaginosa | 1 | 1 (0.69%) | 0 (0%) | 1.000 |
| Staphylococcus_capitis | 1 | 1 (0.69%) | 0 (0%) | 1.000 |
| Streptococcus_parasanguinis | 1 | 1 (0.69%) | 0 (0%) | 1.000 |
| Streptococcus_tigurinus | 1 | 1 (0.69%) | 0 (0%) | 1.000 |
| Tropheryma_whipplei | 1 | 1 (0.69%) | 0 (0%) | 1.000 |
| Haemophilus_parainfluenzae | 1 | 0 (0%) | 1 (1.64%) | 0.298 |
| Mycobacterium_avium | 1 | 0 (0%) | 1 (1.64%) | 0.298 |
| Staphylococcus_epidermidis | 1 | 0 (0%) | 1 (1.64%) | 0.298 |
| Staphylococcus_epidermidis | 1 | 0 (0%) | 1 (1.64%) | 0.298 |
| Streptococcus_pyogenes | 1 | 0 (0%) | 1 (1.64%) | 0.298 |
| Orientia_tsutsugamushi | 1 | 1 (0.69%) | 0 (0.00%) | 1.000 |
| **Fungi** |  |  |  |  |
| Candida_albicans | 37 | 29 (20.14%) | 8 (13.11%) | 0.232 |
| Pneumocystis_jirovecii | 12 | 11 (7.64%) | 1 (1.64%) | 0.114 |
| Candida_tropicalis | 6 | 5 (3.47%) | 1 (1.64%) | 0.672 |
| Candida_glabrata | 5 | 4 (2.78%) | 1 (1.64%) | 1.000 |
| Aspergillus_fumigatus | 2 | 2 (1.39%) | 0 (0.00%) | 1.000 |
| Cryptococcus_neoformans | 2 | 2 (1.39%) | 0 (0.00%) | 1.000 |
| Aspergillus_flavus | 1 | 1 (0.69%) | 0 (0.00%) | 1.000 |
| Aspergillus_niger | 1 | 1 (0.69%) | 0 (0.00%) | 1.000 |
| Candida_orthopsilosis | 1 | 1 (0.69%) | 0 (0.00%) | 1.000 |
| Talaromyces_marneffei | 1 | 0 (0.00%) | 1 (1.64%) | 0.298 |
| **Virus** |  |  |  |  |
| Human_herpesvirus_1 | 25 | 17 (11.81%) | 8 (13.11%) | 0.793 |
| Human_herpesvirus_4 | 16 | 13 (9.03%) | 3 (4.92%) | 0.403 |
| Human_herpesvirus_5 | 16 | 12 (8.33%) | 4 (6.56%) | 0.782 |
| Torque_teno_virus | 7 | 4 (2.78%) | 3 (4.92%) | 0.427 |
| Human_herpesvirus_7 | 4 | 3 (2.08%) | 1 (1.64%) | 1.000 |
| Hepatitis_B_virus | 2 | 2 (1.39%) | 0 (0.00%) | 1.000 |
| Human_herpesvirus_6A | 2 | 2 (1.39%) | 0 (0.00%) | 1.000 |
| Torque_teno_virus_16 | 1 | 1 (0.69%) | 0 (0.00%) | 1.000 |
| BK_polyomavirus | 1 | 0 (0.00%) | 1 (1.64%) | 0.298 |

**Supplementary Table 3.** Comparison of pathogens detected by mNGS between immunocompetent and immunocompromised patients with SCAP.

| **Infected pathogens** | **Total** | **Immunocompetent** | **Immunocompromised** | ***P* value** |
| --- | --- | --- | --- | --- |
|  |  | **(n=121)** | **(n=23)** |  |
| **Bacteria** |  |  |  |  |
| Mycobacterium_tuberculosis | 21 | 19 (15.70%) | 2 (8.70%) | 0.528 |
| Escherichia_coli | 11 | 9 (7.44%) | 2 (8.70%) | 0.689 |
| Klebsiella_pneumoniae | 9 | 8 (6.61%) | 1 (4.35%) | 1.000 |
| Corynebacterium_striatum | 7 | 6 (4.96%) | 1 (4.35%) | 1.000 |
| Mycobacterium_abscessus | 6 | 4 (3.31%) | 2 (8.70%) | 0.245 |
| Acinetobacter_baumannii | 6 | 8 (6.61%) | 0 (0.00%) | 0.356 |
| Chlamydia_psittaci | 5 | 5 (4.13%) | 0 (0.00%) | 1.000 |
| Pseudomonas_aeruginosa | 5 | 5 (4.13%) | 0 (0.00%) | 1.000 |
| Streptococcus_pneumoniae | 4 | 3 (2.48%) | 1 (4.35%) | 0.505 |
| Staphylococcus_haemolyticus | 4 | 4 (3.31%) | 0 (0.00%) | 1.000 |
| Haemophilus_influenzae | 3 | 3 (2.48%) | 0 (0.00%) | 1.000 |
| Legionella_longbeachae | 2 | 1 (0.83%) | 1 (4.35%) | 0.295 |
| Legionella_pneumophila | 2 | 1 (0.83%) | 1 (4.35%) | 0.295 |
| Burkholderia_cenocepacia | 2 | 2 (1.65%) | 0 (0.00%) | 1.000 |
| Chlamydia_abortus | 2 | 2 (1.65%) | 0 (0.00%) | 1.000 |
| Chlamydophila_abortus | 2 | 2 (1.65%) | 0 (0.00%) | 1.000 |
| Enterococcus_faecalis | 2 | 2 (1.65%) | 0 (0.00%) | 1.000 |
| Streptococcus_constellatus | 2 | 2 (1.65%) | 0 (0.00%) | 1.000 |
| Eikenella_corrodens | 1 | 1 (0.83%) | 0 (0.00%) | 1.000 |
| Elizabethkingia_anophelis | 1 | 1 (0.83%) | 0 (0.00%) | 1.000 |
| Elizabethkingia_meningoseptica | 1 | 1 (0.83%) | 0 (0.00%) | 1.000 |
| Enterobacter_cloacae | 1 | 1 (0.83%) | 0 (0.00%) | 1.000 |
| Enterococcus_faecium | 1 | 1 (0.83%) | 0 (0.00%) | 1.000 |
| Fusobacterium_nucleatum | 1 | 1 (0.83%) | 0 (0.00%) | 1.000 |
| Klebsiella_oxytoca | 1 | 1 (0.83%) | 0 (0.00%) | 1.000 |
| Mycobacterium_intracellulare | 1 | 1 (0.83%) | 0 (0.00%) | 1.000 |
| Mycoplasma_hominis | 1 | 1 (0.83%) | 0 (0.00%) | 1.000 |
| Prevotella_intermedia | 1 | 1 (0.83%) | 0 (0.00%) | 1.000 |
| Propionibacterium_acnes | 1 | 1 (0.83%) | 0 (0.00%) | 1.000 |
| Rothia_mucilaginosa | 1 | 1 (0.83%) | 0 (0.00%) | 1.000 |
| Serratia_marcescens | 1 | 1 (0.83%) | 0 (0.00%) | 1.000 |
| Staphylococcus_aureus | 1 | 1 (0.83%) | 0 (0.00%) | 1.000 |
| Stenotrophomonas_maltophilia | 1 | 1 (0.83%) | 0 (0.00%) | 1.000 |
| Streptococcus_parasanguinis | 1 | 1 (0.83%) | 0 (0.00%) | 1.000 |
| Streptococcus_tigurinus | 1 | 1 (0.83%) | 0 (0.00%) | 1.000 |
| Tropheryma_whipplei | 1 | 1 (0.83%) | 0 (0.00%) | 1.000 |
| Mycobacterium_chimaera | 1 | 0 (0.00%) | 1 (4.35%) | 0.160 |
| Staphylococcus_capitis | 1 | 0 (0.00%) | 1 (4.35%) | 0.160 |
| Streptococcus_mitis | 1 | 0 (0.00%) | 1 (4.35%) | 0.160 |
| Orientia_tsutsugamushi | 1 | 1 (0.83%) | 0 (0.00%) | 1.000 |
| **Fungi** |  |  |  |  |
| Candida_albicans | 29 | 25 (20.66%) | 4 (17.39%) | 1.000 |
| Pneumocystis_jirovecii | 11 | 5 (4.13%) | 6 (26.09) | **<0.001** |
| Candida_tropicalis | 5 | 4 (3.31%) | 1 (4.35%) | 0.587 |
| Candida_glabrata | 4 | 4 (3.31%) | 0 (0.00%) | 1.000 |
| Aspergillus_fumigatus | 2 | 1 (0.83%) | 1 (4.35%) | 0.295 |
| Cryptococcus_neoformans | 2 | 1 (0.83%) | 1 (4.35%) | 0.295 |
| Aspergillus_flavus | 1 | 1 (0.83%) | 0 (0.00%) | 1.000 |
| Candida_orthopsilosis | 1 | 1 (0.83%) | 0 (0.00%) | 1.000 |
| Aspergillus_niger | 1 | 0 (0.00%) | 1 (4.35%) | 0.160 |
| **Virus** |  |  |  |  |
| Human_herpesvirus_1 | 17 | 14 (11.57%) | 3 (13.04%) | 0.736 |
| Human_herpesvirus_4 | 13 | 10 (8.26%) | 3 (13.04%) | 0.437 |
| Human_herpesvirus_5 | 12 | 10 (8.26%) | 2 (8.70%) | 1.000 |
| Torque_teno_virus | 4 | 3 (2.48%) | 1 (4.35%) | 0.505 |
| Human_herpesvirus_7 | 3 | 2 (1.65%) | 1 (4.35%) | 0.409 |
| Hepatitis_B_virus | 2 | 1 (0.83%) | 1 (4.35%) | 0.295 |
| Human_herpesvirus_6A | 2 | 2 (1.65%) | 0 (0.00%) | 1.000 |
| Torque_teno_virus_16 | 1 | 0 (0.00%) | 1 (4.35%) | 0.160 |
